# Students in Philadelphia

**Published:** 1859-05

**Authors:** 


STUDENTS IN PHILADELPHIA.—We have every reason to believe that the medical classes in this city were larger during the past session than in any other city in the world. The number was 1163, an increase of 24 over the session of 1857-58. The graduating classes were also larger, showing 451 against 407 of the previous year.

